# HuR Is Necessary for Mammary Epithelial Cell Proliferation and Polarity at Least in Part via ΔNp63

**DOI:** 10.1371/journal.pone.0045336

**Published:** 2012-09-18

**Authors:** Wensheng Yan, Yanhong Zhang, Jin Zhang, Seong-Jun Cho, Xinbin Chen

**Affiliations:** Comparative Oncology Laboratory, University of California Davis, Davis, California, United States of America; Garvan Institute of Medical Research, Australia

## Abstract

HuR, a RNA binding protein, is known to function as a tumor maintenance gene in breast cancer and associated with tumor growth and poor prognosis. However, the cellular function of this protein remains largely unknown in normal mammary epithelial cells. Here, we showed that in immortalized MCF10A mammary epithelial cells, HuR knockdown inhibits cell proliferation and enhances premature senescence. We also showed that in three-dimensional culture, MCF10A cells with HuR knockdown form abnormal acini with filled lumen and an aberrant expression pattern of the extracellular matrix protein laminin V. In addition, we showed that HuR knockdown increases ΔNp63, but decreases wild-type p53, expression in MCF10A cells. Moreover, we showed that ΔNp63 knockdown partially rescues the proliferative defect induced by HuR knockdown in MCF10A cells. Consistent with this, we identified two U-rich elements in the 3′-untranslated region of p63 mRNA, to which HuR specifically binds. Finally, we showed that HuR knockdown enhances ΔNp63 mRNA translation but has no effect on p63 mRNA turnover. Together, our data suggest that HuR maintains cell proliferation and polarity of mammary epithelial cells at least in part via ΔNp63.

## Introduction

Posttranscriptional regulation, an important process in the control of gene expression, starts with interactions of RNA-binding proteins with cis-acting elements in the regulated transcripts [1], [2]. HuR is among the most prominent RNA binding proteins, which modulates mRNA stability and translation, and consequently regulates cell proliferation, angiogenesis, apoptosis, and stress response.

HuR, a member of the Hu family, is ubiquitously expressed and related to Drosophila embryonic lethal abnormal vision protein [3]. The other three members of the Hu family, HuB/HelN1, HuC and HuD, are primarily expressed in the neuronal tissues [4]. HuR contains three RNA-recognition motifs through which it binds to AU- or U-rich sequences in 3′-untranslated regions (3′UTR) of target mRNAs [5]. HuR is predominantly localized in the nucleus under non-stress conditions. Upon stimulation, such as heat shock, HuR is exported to cytoplasm where it regulates mRNA stability and/or translation [6]. The export of HuR is mediated at least by two pathways, transporting by transportins 1 and 2 [7], or by pp32 and APRIL in CRM1-dependent manner [6].

To date, elevated expression of HuR is associated with carcinogenesis in a wide variety of human tumors, including breast, colon, and prostate [8], [9], [10]. High levels of cytoplasmic HuR are associated with poor differentiation, large tumor size, and short survival in patients with breast ductal carcinoma [11] and non-BRCA1/2 mutated hereditary breast cancer [12]. The biological function of HuR in breast cancer is dependent on the mRNAs to which it is binding [4], [13]. Elevated cytoplasmic HuR in breast cancer cells increases cyclin E1 and COX-2 expression and growth potential of cancer cells [8], [14]. In addition, ectopic expression of HuR decreases BRCA1 expression [15]. In invasive breast tumors, HuR suppresses Wnt-5a mRNA translation [16], and reduced Wnt-5a expression is known to shorten disease-free survival [17]. Interestingly, miR-125a decreases HuR protein translation in breast cancer cells, and consequently inhibits cell proliferation and promotes apoptosis [18]. As such, HuR is established as a marker for breast cancer aggressiveness and poor prognosis as well as a target for treating breast cancer. Thus, delineation of HuR function in normal mammary epithelial cells is warranted.

P63 is known to be pivotal for the development and maintenance of epithelial tissues. *p63^−/−^* mice display gross developmental abnormalities. The most striking defect is complete lack of all stratified epithelia and their derivatives, including epidermis and mammary glands [19]. Recently, we showed that p63 mRNA stability is regulated by RNPC1, a RNA-binding protein, via AU-/U-rich elements in p63 3′ UTR [20]. Considering that HuR prefers to bind to AU-/U-rich elements in 3′ UTR of its targets, we explored whether HuR regulates p63 expression and cell proliferation in mammary breast epithelial cells.

## Results

### HuR Knockdown Inhibits Proliferation of Normal Mammary Epithelial Cells

**Figure 1:**
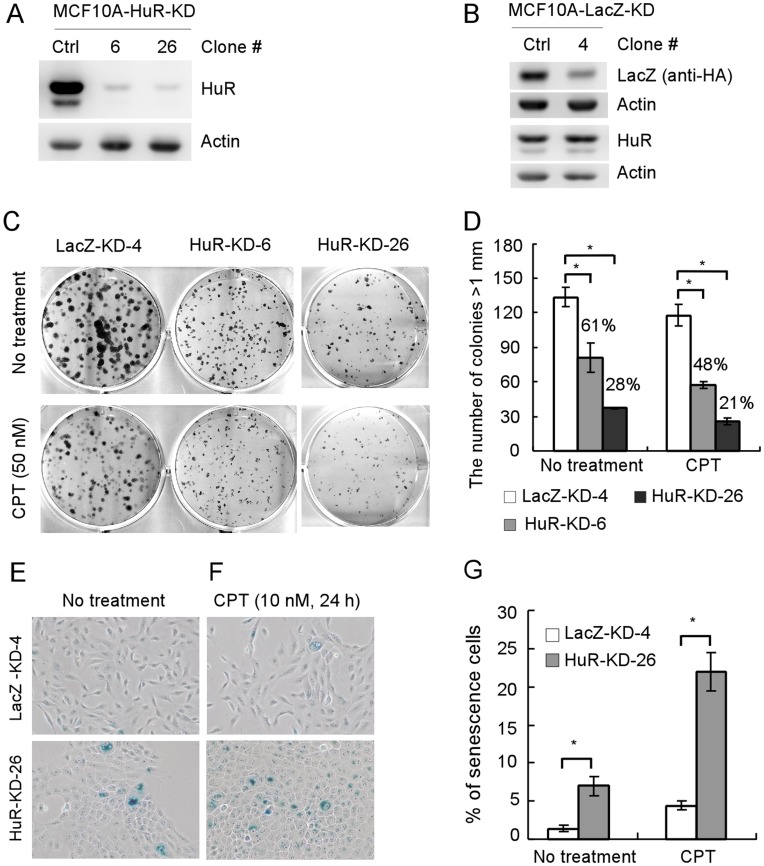
HuR Knockdown inhibits proliferation of mammary epithelial cells. (A) Generation of MCF10A cell lines in which HuR can be stably knocked down. Western blots were prepared with extracts from MCF10A cells with or without stable HuR knockdown, and then probed with antibodies against HuR and actin, respectively. (B) Generation of MCF10A-LacZ-KD cell line. Western blots were prepared with extracts from parental MCF10A cells or MCF10A-LacZ-KD cells, in which pcDNA3-HA-LacZ plasmid was transiently transfected for 24 h, and then probed with antibodies against HA, HuR and actin, respectively. (C) Colony formation assay was preformed with MCF10A-LacZ-KD or MCF10A-HuR-KD cells treated with or without 50 nM camptothecin for 4 h at day 3. (D) Quantification of colonies shown in (C) from three separate experiments. Asterisk indicates a significant difference (*P<*0.05). (E) SA-β-galactosidase staining assay was performed using MCF10A-LacZ-KD or MCF10A-HuR-KD cells. (F) SA-β-galactosidase staining assay was performed as in (E) except that the cells were treated with 10 nM camptothecin for 24 h. (G) Quantification of the percentage of SA-β-galactosidase-positive cells as shown in (E-F) from three separate experiments. Asterisk indicates a significant difference (*P<*0.05).

Several studies have been performed to examine HuR function in breast tumor tissues and cell lines. These study showed that HuR regulates multiple pathways involved in breast carcinoma formation [8], [11], [12], [14], [15], [21]. However, these systems are relatively intractable for studying HuR function in normal mammary epithelial cells. MCF10A is a spontaneously immortalized, but nontransformed human mammary epithelial cell line [22]. This cell line exhibits features of normal mammary epithelium, such as lack of tumorigenicity in nude mice and requirement of multiple growth factors and hormones for proliferation and survival [22]. Importantly, MCF10A cells form acinar structures in three-dimensional culture, a characteristic of normal glandular epithelium *in vivo* [23], [24]. Furthermore, ectopic expression of oncogenes in MCF10A cells, such as mutant p53 and ErbB2, disrupts this morphogenetic process [25], [26]. Thus, in this study, MCF10A cells were used to address the implication of HuR in normal mammary epithelial cells. First, we generated multiple MCF10A cells in which HuR can be stably knocked down. Two representative HuR-KD MCF10A cell lines were shown in Fig. 1A (no. 6 and 26). We found that the level of HuR protein was significantly reduced in MCF10A cells with HuR knockdown. The level of actin protein was examined as a loading control. As a negative control, LacZ-knockdown MCF10A cell line was generated (Fig. 1B, no. 4). Next, to examine whether HuR knockdown has any effect on cell proliferation, MCF10A-HuR-KD cell lines no. 6 and 26 were used for long-term colony formation assay. We found that HuR knockdown markedly inhibited cell proliferation because the numbers of colonies formed by MCF10A-HuR-KD cell lines no. 6 and 26 were reduced to 61% and 28% of the control cells, respectively (Fig. 1C, top panel, and 1D). Camptothecin, an inhibitor of DNA topoisomerase I, is known to inhibit cell proliferation and induce senescence in low concentration [27], [28]. Thus, we tested whether HuR knockdown made MCF10A cells more sensitive to camptothecin treatment. We found that MCF10A cells with HuR knockdown were highly sensitive to short-term treatment of 50 nM camptothecin because the numbers of colonies formed by MCF10A-HuR-KD cell lines no. 6 and 26 were reduced to 48% and 21% of the control cells, respectively (Fig. 1C, bottom panel, and 1D). To further confirm the requirement of HuR for proliferation of mammary epithelial cells, we found that HuR knockdown promoted premature senescence in MCF10A cells (Fig. 1E and 1G). In addition, HuR knockdown sensitized MCF10A cells to camptothecin-induced premature senescence (Fig. 1F–G). This result suggests that HuR is necessary for normal cell proliferation of MCF10A mammary epithelial cells.

### HuR Knockdown Disrupts the Formation of Polarized Acinus-like Architecture

**Figure 2:**
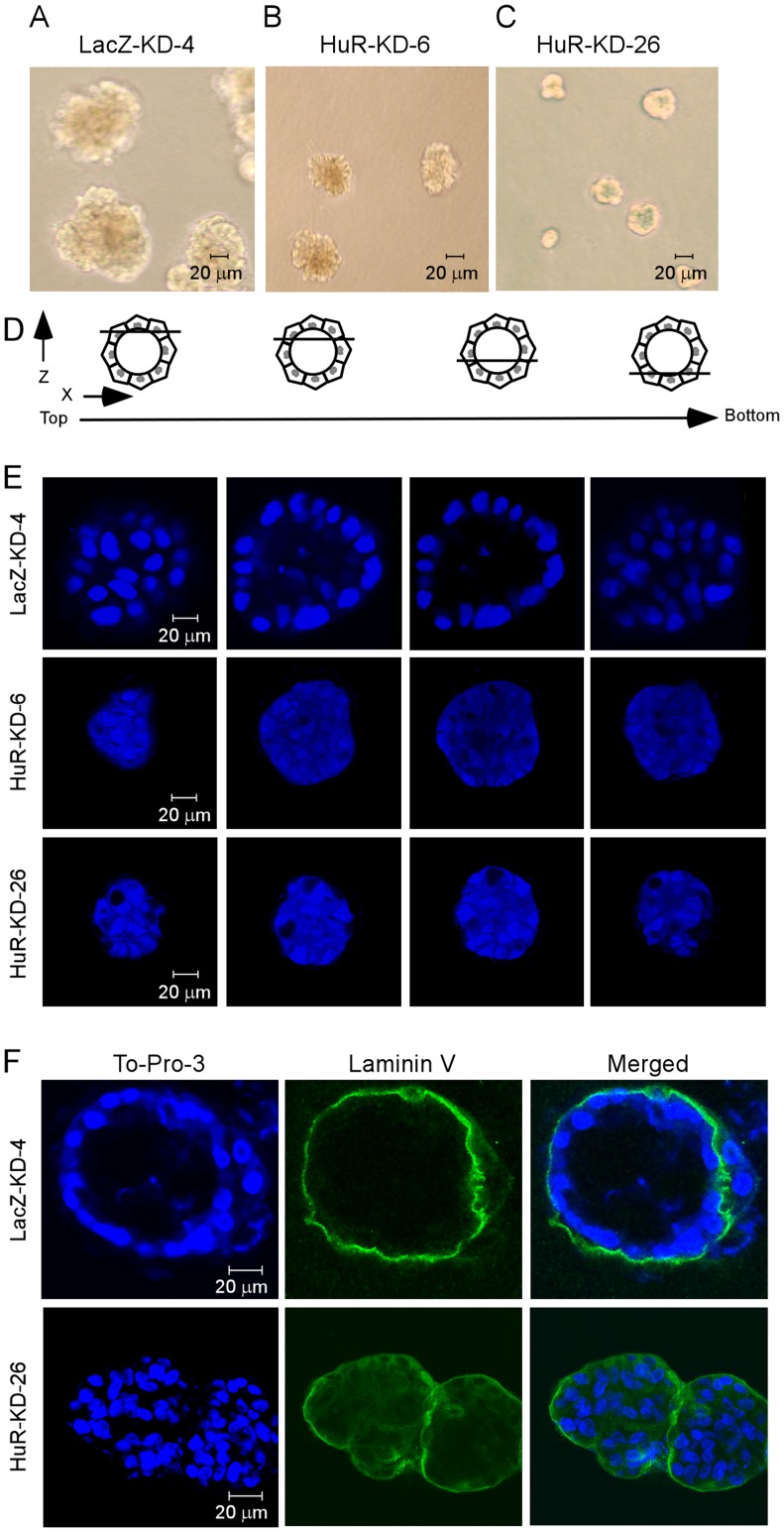
HuR Knockdown disrupts the formation of acinar-like architecture in mammary epithelial cells. (A–C) Representative phase-contrast microscopic images of MCF10A-LacZ-KD (A) and MCF10A-HuR-KD (B-C) cells in 3-D culture from three separate experiments. All 100×, scale bar, 20 µm. (D) Schematic diagrams of serial confocal cross-sections (x–y axis) through a hypothetical MCF10A acinus. The diagrams overlaying each section illustrate the relative position of the optical section with respect to the *z* axis. (E) Serial confocal cross-sections of an acinus from MCF10A-LacZ-KD (top panel) or MCF10A-HuR-KD (middle and bottom panels) cells. The cells were cultured on Matrigel for 19 days. To-Pro-3 was used for nucleus staining. All 250×, scale bar, 20 µm. Experiments were performed in triplicates. (F) Immunostaining of laminin V in 3-D structures formed by MCF10A-LacZ-KD-4 (top panel) or MCF10A-HuR-KD-26 (bottom panels) cells. Laminin V is normally deposited at the basal surface of the spheroid. All 250×, scale bar, 20 µm.

To determine whether HuR knockdown regulates cell polarity, we examined acinar structures of MCF10A cells in 3-D culture. We found that compared to control cells (Fig. 2A), MCF10A cells with HuR knockdown formed smaller acinar structures (Fig. 2B–C). In addition, we found that control LacZ-KD MCF10A cells formed polarized acinar architectures along with hollow lumen (Fig. 2E, top), consistent with previous reports [23], [29], [30]. However, MCF10A cells with HuR knockdown formed an acinus with filled lumen (Fig. 2E, middle and bottom).

To further demonstrate that HuR is required for the normal architecture of MCF10A acini, we immunostained laminin V, a principal extracellular matrix protein, in acini formed by MCF10A-LacZ-KD-4 and MCF10A-HuR-KD-26 cells in 3-D culture. We found that laminin V was deposited at the basal surface of the spheroid formed by MCF10A-LacZ-KD-4 cells (Fig. 2F, top). In contrast, in MCF10A-HuR-KD-26 cells, laminin V was secreted into the lumen of the acini and cell-cell junctions (Fig. 2F, bottom). This result suggests that HuR is required for development of a polarized acinus-like architecture in 3-D culture of MCF10A cells.

### HuR Knockdown Increases ΔNp63, but Decrease Wild-type p53, Expression in MCF10A Cells

**Figure 3:**
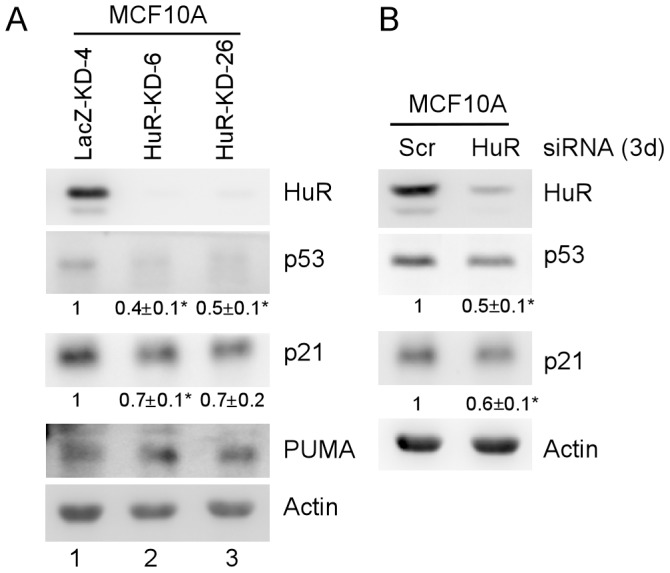
HuR knockdown moderately decreases p53 expression in MCF10A cells. (A) Western blots were prepared using extracts from MCF10A-LacZ-KD (lane 1) and MCF10A-HuR-KD (lanes 2 and 3) cells. The blots were probed with antibodies against HuR, p53, p21, PUMA, and actin, respectively. Experiments were performed in triplicates. The basal levels of p53 and p21 were arbitrarily set at 1.0 and the fold change was shown below each lane. Asterisk indicates a significant difference (*P<*0.05). (B) Western blots were prepared using extracts from MCF10A cells transiently transfected with scrambled siRNA or siRNA to knock down HuR for 3 days, and then probed with antibodies against HuR, p53, p21, and actin, respectively. Quantification and statistical analysis were performed as in (A).

It is well-known that p53 plays a pivotal role in cell proliferation and premature senescence [31], and MCF10A cells carry a wild-type p53 [25]. Therefore, we tested whether p53 is correlated with premature senescence and deficient proliferation in MCF10A cells with HuR knockdown. We found that compared to control cells, the level of p53 protein was moderately decreased in MCF10A cells with HuR knockdown (Fig. 3A). This was consistent with previous reports that HuR contributes to induction of p53 expression via direct association with AU-rich element in 3′ UTR of p53 mRNA [32], [33]. Likewise, HuR knockdown slightly decreased the protein level of p21, the transcript of which is also a target of HuR [34]. However, HuR knockdown had little if any effect on PUMA expression in MCF10A cells (Fig. 3A). These results were further confirmed in MCF10A with transient HuR knockdown (Fig. 3B). Thus, due to lack of correlation between cell proliferation and levels of p53 and p21, we conclude that both p53 and p21 are unlikely to play a role in premature senescence and deficient cell proliferation in MCF10A cells with HuR knockdown.

**Figure 4:**
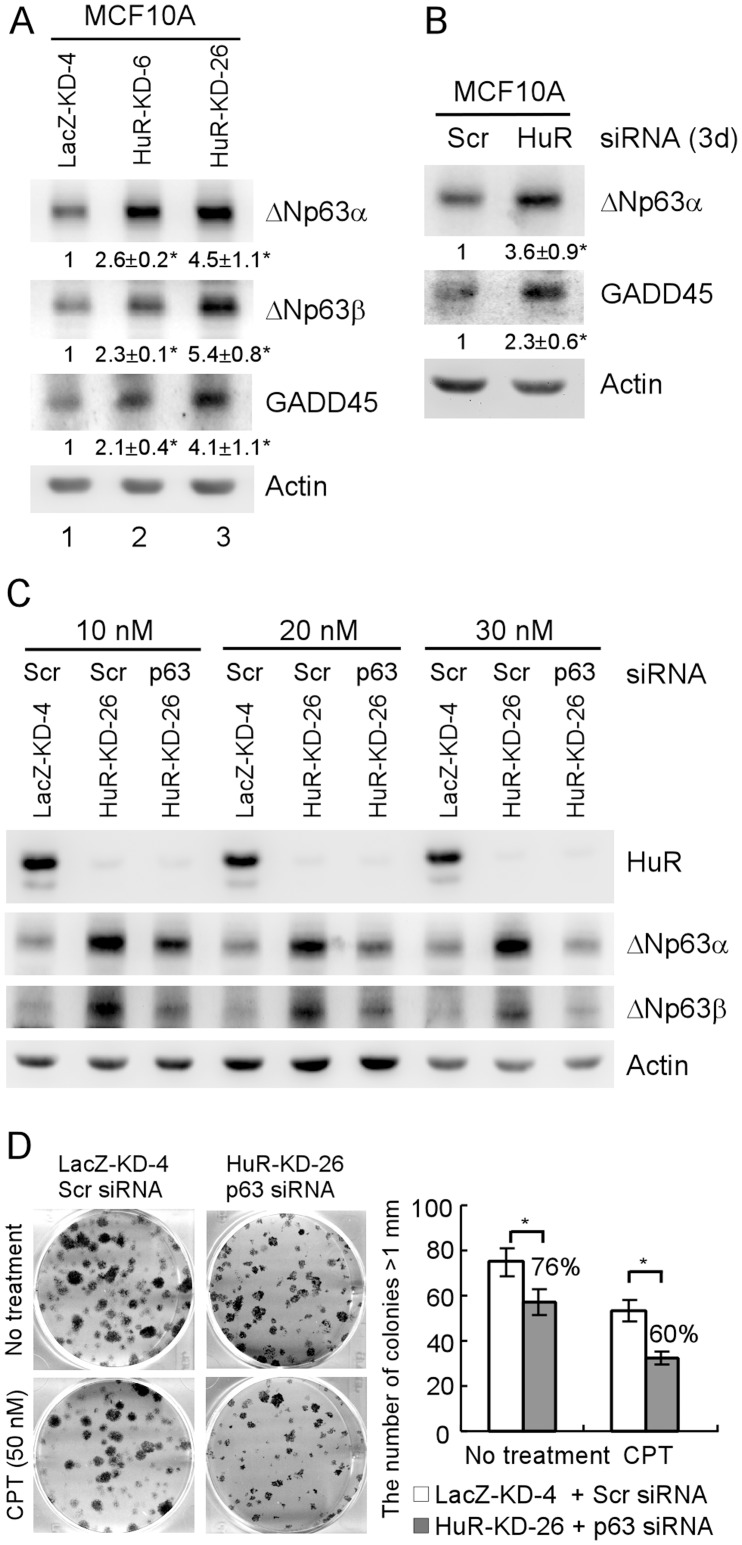
HuR knockdown increases ΔNp63 expression in MCF10A cells. (A) Western blots were prepared using extracts from MCF10A-LacZ-KD cells (lane 1) and MCF10A-HuR-KD cells (lanes 2 and 3). The blots were probed with antibodies against p63, GADD45, and actin, respectively. Experiments were performed in triplicates. The basal levels of ΔNp63α, ΔNp63β, and GADD45 were arbitrarily set at 1.0 and the fold change was shown below each lane. Asterisk indicates a significant difference (*P<*0.05). (B) Western blots were prepared using extracts from MCF10A cells transiently transfected with scrambled siRNA or siRNA to knock down HuR for 3 days, and then probed with antibodies against p63, GADD45, and actin, respectively. Quantification and statistical analysis were performed as in (A). (C) Western blots were prepared using extracts from MCF10A-LacZ-KD-4 and MCF10A-HuR-KD-26 cells, which were transiently transfected with various concentrations of scrambled siRNA or p63 siRNA for 3 days, and then probed with antibodies against HuR, p63, and actin, respectively. (D) Left panel, colony formation assay was preformed with MCF10A-LacZ-KD-4 and MCF10A-HuR-KD-26 cells, which were transfected with 30 nM of scrambled siRNA and p63 siRNA, respectively. After 1 day, the cells was split into 6-well plates, and then treated with or without 50 nM camptothecin for 4 h at day 2. Right panel, quantification of colonies shown in left panel from three separate experiments. Asterisk indicates a significant difference (*P<*0.05).

Previously, we showed that ΔNp63 isoforms, especially ΔNp63β, possess a remarkable ability to transactivate target genes and suppress cell proliferation [35], [36]. MCF10A cells express a high level of ΔNp63 [23]. Thus, we examined whether ΔNp63 plays a role in HuR-mediated premature senescence and cell proliferation. We found that the protein levels of ΔNp63α and ΔNp63β were increased in MCF10A cells with HuR knockdown (Fig. 4A). Interestingly, we found that expression of GADD45, a target of ΔNp63 and a mediator for growth suppression, was also increased in MCF10A cells upon HuR knockdown (Fig. 4A). Consistent with this, we found that transient HuR knockdown obviously increased the expression of ΔNp63 and GADD45 in MCF10A cells (Fig. 4B).

To assess whether ΔNp63 is sufficient to mediate HuR function in cell proliferation, we tested several concentrations of p63 siRNA to counteract the upregulation of ΔNp63 induced by HuR knockdown in MCF10A-HuR-KD-26 cells. We found that 30 nM p63 siRNA efficiently reduced the levels of ΔNp63α and ΔNp63β proteins in MCF10A-HuR-KD-26 cells to the basal levels of ΔNp63α and ΔNp63β proteins in control cells (Fig. 4C). Thus, 30 nM p63 siRNA was chosen for colony formation assay in MCF10A-HuR-KD-26 cells. We found that ΔNp63 knockdown partially rescued the proliferative defect induced by HuR knockdown in MCF10A-HuR-KD-26 cells. The number of colonies formed by MCF10A-HuR-KD-26 cells with ΔNp63 knockdwon was only reduced to 76% of the control cells (Fig. 4D), compared to 28% in MCF10A-HuR-KD-26 cells without ΔNp63 knockdwon (Fig. 1C–D). Furthermore, we found that ΔNp63 knockdwon made MCF10A-HuR-KD-26 cells resistant to camptothecin treatment. The number of colonies was only reduced to 60% of the control cells (Fig. 4D), compared to 20% in MCF10A-HuR-KD-26 cells without ΔNp63 knockdwon (Fig. 1C–D). Together, these data suggest that ΔNp63, but not p53, may at least in part mediate HuR-knockdown-induced growth suppression and premature senescence in MCF10A mammary epithelial cells.

### HuR Binds to U-rich Elements in 3′UTR of ΔNp63 Transcript

**Figure 5:**
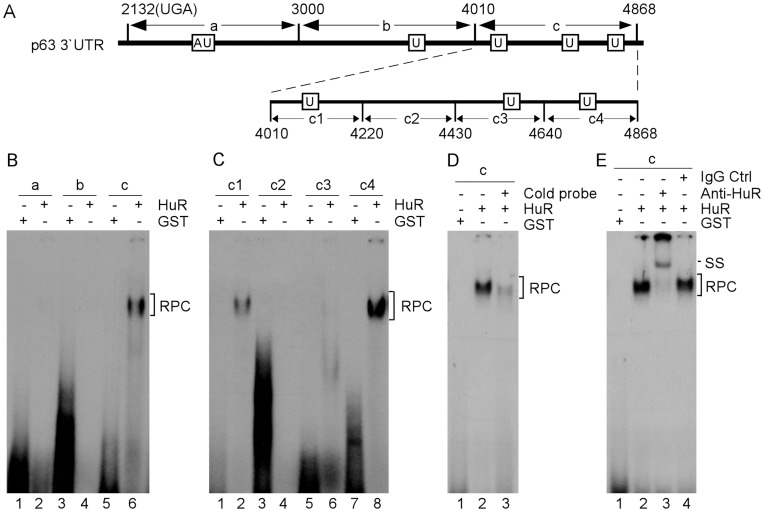
HuR binds to U-rich elements in 3′UTR of ΔNp63 transcript. (A) Schematic presentation of p63 3′UTR and probes used for REMSA. (B) REMSA was performed by mixing ^32^P-labeled RNA probe a, b, or c with recombinant GST or GST-fused HuR. The bracket indicates RNA-protein complexes. (C) REMSA was performed by mixing ^32^P-labeled RNA probe c1, c2, c3, or c4 with recombinant GST or GST-fused HuR. The bracket indicates RNA-protein complexes. (D) For competition assay, an excess amount of unlabeled p21 cold probe was added to a reaction mixture containing HuR and probe c. The bracket indicates RNA-protein complexes. (E) For supershift assay, 3 µg of control IgG or anti-HuR was added to a reaction mixture containing probe c with or without GST-HuR.

It is known that HuR functions as a RNA binding protein and binds to AU-/U-rich elements in 3′ UTRs of its target mRNAs [5]. Thus, we explored whether HuR regulates ΔNp63 expression via directly binding to its transcript. For this purpose, RNA electrophoretic mobility shift assay (REMSA) was performed to identify potential HuR binding regions in ΔNp63 transcript. First, we mixed recombinant GST or GST-fused HuR with ^32^P-labeled probes a, b, or c, which cover the entire 3′ UTR of ΔNp63 transcript (Fig. 5A). We found that GST-fused HuR, but not GST protein, formed a complex with probe c (Fig. 5B). Next, to delineate the binding site of HuR in ΔNp63 3′UTR, probes c1-c4 were generated for REMSA (Fig. 5A). We found that HuR fusion protein strongly bound to probes c1 and c4, but not c2 and c3 (Fig. 5C, compare lanes 1, 3, 5, and 7 with lanes 2, 4, 6, and 8, respectively). Furthermore, the binding specificity of HuR to probe c was examined in competition assay. We found that the probe c-HuR complex was inhibited by an excess amount of cold p21 probe (Fig. 5D, lane 3). P21 3′UTR is known to contain a HuR binding site [34]. In addition, the probe c-HuR complex was super-shifted by anti-HuR antibody (Fig. 5E, lane 3), but not by control IgG (Fig. 5E, lane 4). These data suggest that the U-rich elements located within nt 4010–4220 and nt 4640–4868 in 3′UTR of ΔNp63 transcript are required for HuR to regulate ΔNp63 expression.

### HuR Knockdown Enhances ΔNp63 mRNA Translation but has no Effect on mRNA Turnover

**Figure 6:**
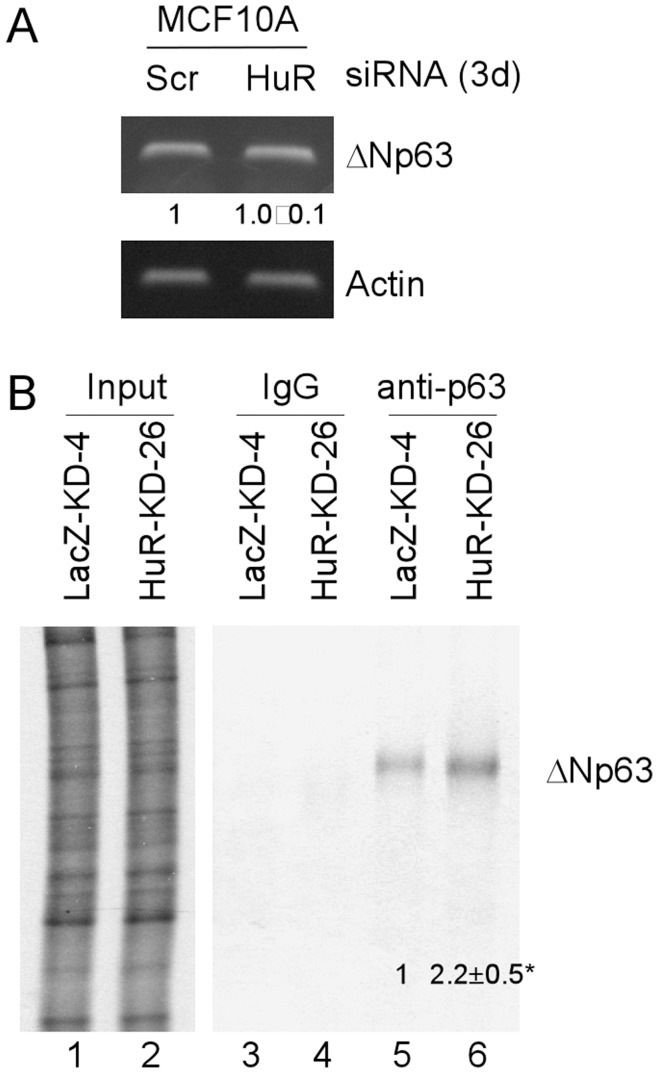
HuR Knockdown enhances ΔNp63 mRNA translation without affecting mRNA turnover. (A) RT-PCR was performed with total RNAs isolated from MCF10A cells transiently transfected with scrambled siRNA or siRNA to knock down HuR for 3 days. (B) HuR knockdown increased the newly synthesized ΔNp63 protein. Cell extracts were prepared from MCF10A-LacZ-KD or MCF10A-HuR-KD cells labeled with [^35^S]methionine for 30 min. The levels of ^35^S-labeled proteins were measured by scintillation counter and an equal amount of ^35^S-labeled proteins was used for immunoprecipitation. ^35^S-labeled ΔNp63 protein was immunoprecipitated with mouse anti-p63 monoclonal antibody or a control IgG and visualized by autoradiography. Input was used as a loading control. Experiments were performed in triplicates. The level of ΔNp63α in MCF10A-LacZ-KD-4 cells was arbitrarily set at 1.0 and the fold change in MCF10A-HuR-KD-26 cells was shown below the lane.

HuR protein is best-known for its function in the regulation of mRNA stability. Thus, we explored whether HuR knockdown has an effect on the level of ΔNp63 mRNA. We found that the level of ΔNp63 mRNA was not significantly altered by HuR knockdown in MCF10A cells (Fig. 6A). HuR protein is also known to suppress translation of its targets without affecting mRNA turnover, such as Wnt-5a [16] and BRCA1 [15]. Thus, we examined whether HuR knockdown increases the rate of ΔNp63 mRNA translation. To test this, we labeled newly synthesized ΔNp63 protein with ^35^S-methionine for a short period of 30 min in MCF10A-LacZ-KD and MCF10A-HuR-KD cells. We found that the level of newly synthesized ΔNp63 protein was obviously increased in MCF10A cells with HuR knockdown (Fig. 6B, compare lane 5 with 6). Together, our data suggest that HuR regulates ΔNp63 expression via mRNA translation in MCF10A cells.

## Discussion

Here, we found that in MCF10A cells, HuR knockdown inhibits cell proliferation and enhances premature senescence. In addition, we found that in 3-D culture, MCF10A cells with HuR knockdown develop abnormal acinar architectures. Furthermore, we showed that HuR knockdown increases ΔNp63, but decreases wild-type p53, expression in the MCF10A cells. Correspondingly, we showed that ΔNp63 knockdown partially rescues the proliferative defect induced by HuR knockdown in MCF10A cells. Consistent with this, we found that HuR can specifically bind to two U-rich regions in 3′ UTR of p63 mRNA and consequently inhibits ΔNp63 expression via translation. Thus, our data suggest that HuR is necessary for maintaining cell proliferation and polarity of MCF10A cells at least in part via regulating ΔNp63 expression.

p63, a member of the p53 family, is expressed as two major groups, the TA and ΔN variants [37], [38]. The TA variant, which is expressed from the upstream promoter, contains an activation domain similar to that in p53 [38]. Thus, TAp63 has a strong transcriptional activity and is capable of inducing cell cycle arrest and apoptosis when overexpressed [37], [38]. In contrast, the ΔNp63 variant, which is expressed from a promoter in intron 3, lacks such an activation domain [38] but obtains 14 unique residues at N terminus. These 14 amino acids together with the adjacent proline-rich region constitute an activation domain for the ΔN variant [35]. ΔNp63 isoforms have been highlighted to possess oncogenic potential and act as dominant-negative molecules against both TAp63 isoforms and p53 [38]. However, many studies also suggest that ΔNp63 acts as a tumor suppressor. ΔNp63, especially ΔNp63β, possesses a remarkable ability to transactivate target genes and suppress cell proliferation [35], [36]. In mammary progenitor cells, knockdown of ΔNp63 results in genomic instability and increased cell proliferation [39]. In addition, depletion of both ΔNp63α and ΔNp63β results in epithelial to mesenchymal transition in MCF10A cells, which can be rescued by expression of ΔNp63α [40]. Consistent with this, ΔNp63 expression is found to be repressed in breast tumors [41]. Studies showed that ΔNp63 is the predominant isoform in MCF10A mammary epithelial cells, whereas TAp63s are expressed at very low levels and undetectable by immunoblot [23], [42]. Here, we found that HuR knockdown obviously increases ΔNp63, but moderately decreases wild-type p53, expression in MCF10A cells. In addition, knockdown of both ΔNp63α and ΔNp63β partially rescues the proliferative defect induced by HuR knockdown in MCF10A cells. Thus, upregulation of ΔNp63, especially ΔNp63β, in MCF10A mammary epithelial cells may actually transactivate growth-suppressing genes, such as GADD45, to suppress cell proliferation, rather than act as dominant-negative molecules against TAp63 and p53.

HuR has numerous functions mostly related to cell stress response. However, emerging evidence showed that HuR plays a role in the processes of differentiation and development, such as lung branching morphogenesis, placental branching morphogenesis, spermatogenesis, myogenesis and adipogenesis [43], [44], [45], [46], [47]. In this study, we found that in 3-D culture, MCF10A cells with HuR knockdown formed an abnormal acinus with filled lumen and an aberrant expression pattern of laminin V, suggesting that HuR is required for development of a polarized acinus-like architecture in mammary epithelial cells. This effect of HuR knockdown on acinus formation is at least in part mediated by increased ΔNp63 expression, because p63 has an essential role in epithelial development [19], [48]. *p63^−/−^* mice lose all stratified epithelia and their derivatives, including epidermis and mammary glands [19]. Significantly, germline p63 mutations in human are also associated with similar developmental syndromes [49]. In addition, p63 is a key regulator of cell adhesion in mammary epithelial cells. Down- or up-regulation of ΔNp63 caused a profound dysregulation of adhesion-related genes [42], [50]. Thus, the ability of p63 to regulate matrix adhesion could play an important role in maintenance of polarity and integrity of mammary epithelial cells and tissues [51]. However, we can not exclude possibility that other pathways are also involved in formation of abnormal acini in HuR-knockdown MCF10A cells, since HuR regulates other targets [5].

HuR is capable of enhancing or suppressing mRNA translation, such as p53 [32], cytochrome c [52], Wnt-5a [16] and BRCA1 [15]. In this study, we found that HuR binds to two U-rich elements in p63 3′ UTR and inhibits p63 mNRA translation. However, the precise mechanism is still not understood. Recently, we showed that p63 mRNA stability can be regulated by RNPC1, a RNA-binding protein, which specifically bound to AU-/U-rich elements in p63 3′ UTR [20]. Considering that both HuR and RNPC1 prefer to bind to U-rich elements in the distal region of p63 3′ UTR, it is possible that HuR may cooperate with other RNA binding proteins, such as RNPC1, to regulated p63 expression at different posttranscriptional levels. Thus, future studies are warranted to explore how HuR regulates p63 expression by modulating translation complexes or cooperating with other RNA binding proteins.

## Materials and Methods

### Reagents

Growth factor-reduced Matrigel was purchased from BD (Franklin Lakes, NJ). Donor horse serum, DMEM/F12 media and To-Pro-3 were purchased from Invitrogen (Carlsbad, CA). Recombinant human epidermal growth factor was purchased from Peprotech (Rocky Hill, NJ). Hydrocortisone, insulin and cholera toxin were purchased from Sigma (St. Louis, MI). Rabbit anti-p53(FL-393), mouse anti-p63(4A4), rabbit anti-GADD45α(C-20) and rabbit anti-p21(C-19) were purchased from Santa Cruz Biotechnology Inc (Santa Cruz, CA). Rabbit anti-PUMA was purchased from ProSci Incorporated (Poway, CA). Rabbit anti-actin was purchased from Sigma (St. Louis, MI). Mouse Anti-laminin V (D4B5) antibody was purchased from Millipore (Temecula, CA). A small interference RNA (siRNA) against HuR, 5′-GGG AUA AAG UAG CAG GAC A dTdT-3′, a siRNA against p63, 5′-CGA CAG UCU UGU ACA AUU U dTdT-3′, and a scrambled siRNA, 5′-GCA GUG UCU CCA CGU ACU A dTdT-3′, were purchased from Dharmacon RNA Technologies (Chicago, IL).

To generate a construct expressing HuR short hairpin RNA (shRNA), one pair of oligonucleotides (sense, 5′-TCG AGG TCC GGG ATA AAG TAG CAG GAC ATT CAA GAG ATG TCC TGC TAC TTT ATC CCT TTT TG-3′, and antisense, 5′-GAT CCA AAA AGG GAT AAA GTA GCA GGA CAT CTC TTG AAT GTC CTG CTA CTT TAT CCC GGA CC-3′; shRNA targeting region is underlined) was synthesized and cloned into pBabe-U6 at *Bam*H1 and *Xho*1 sites. The plasmid is designated pBabe-U6-shHuR. Similarly, a construct expressing LacZ shRNA, pBabe-U6-shLacZ, was generated with one pair of oligonucleotides (sense, 5′-TCG AGG TCC TTT AAC CGC CAG TCA GGC TTT CAA GAG AAG CCT GAC TGG CGG TTA AAT TTT TG-3′, and antisense, 5′-GAT CCA AAA ATT TAA CCG CCA GTC AGG CTT CTC TTG AAA GCC TGA CTG GCG GTT AAAGGA CC-3′; shRNA targeting region is underlined).

To generate a construct expressing truncated LacZ protein, an 1,163-bp DNA fragment encoding HA-tag and C-terminal 375 amino acids of LacZ (GenBank TM NC_008563) was amplified with forward primer, 5′-AAG CTT ACC ATG TAC CCA TAC GAC GTA CCA GAT TAC GCT GAG TTC CTG CAC TGG ATG G-3′, and reverse primer, 5′-CTC GAG TTA TTT TTG ACA CCA GAC CAA C-3′. The fragment was confirmed by sequencing and then cloned into pcDNA3 at *Hind*III and *Xho*I sites, and the resulting plasmid was designated pcDNA3-HA-LacZ.

### Cell Culture

MCF10A cell line was obtained from American Type Culture Collection (Manassas, VA). The medium for MCF10A cells is composed of DMEM/F12 supplemented with 5% donor horse serum, 20 ng/mL of epidermal growth factor, 10 µg/mL of insulin, 0.5 µg/mL of hydrocortisone, and 100 ng/mL of cholera toxin. Overlay 3-D culture was carried out based on the method described previously with some modifications [23]. Briefly, 4-well chamber slides (Millipore Corporation, Danvers, MA) were pre-coated with 80 µl Matrigel. Single cell suspension was plated onto Matrigel-coated chamber slides at 3500 cells/well in complete growth medium with 2% Matrigel and allowed to grow for 19 days. The medium containing 2% Matrigel was refreshened every 4 days.

To generate stable HuR knockdown cell lines, pBabe-U6-shHuR was transfected into MCF10A cells. HuR knockdown cell lines were selected with puromycin and confirmed by Western blot analysis. The positive cell lines were designated MCF10A-HuR-KD.

To generate the control cell lines expressing LacZ shRNA, pBabe-U6-shLacZ was transfected into MCF10A cells. Puromycin-resistent cell lines were selected, and then transiently transfected with pcDNA3-HA-LacZ. The cell lines expressing LacZ shRNA were confirmed by Western blot analysis with anti-HA antibody. The positive cell lines were designated MCF10A-LacZ-KD.

### RT-PCR Assay

Total RNAs were isolated with Trizol reagent (Invitrogen). cDNAs were synthesized using Iscript™ cDNA synthesis kit (Bio-Rad). To measure the level of ΔNp63 mRNA, RT-PCR was performed with forward primer 5′-TGG CAA AAT CCT GGA GCC AG-3′ and reverse primer 5′-GTC TGT GTT ATA GGG ACT GG-3′. Actin mRNA was amplified with forward primer 5′-TCC ATC ATG AAG TGT GAC GT-3′ and reverse primer 5′-TGA TCC ACA TCT GCT GGA AG-3′.

### Colony Formation Assay

To determine whether HuR knockdown affects the proliferation of MCF10A cells, MCF10A-HuR-KD or MCF10A-LacZ-KD cells (600 cells/+well) were cultured in 6-well plates for the 9 days, and then fixed with methanol/glacial acetic acid (7∶1) and stained with 0.1% of crystal violet.

### Confocal Microscopy

The 3-D structures in Matrigel were fixed with 4% paraformaldehyde at room temperature for 20 min. The nuclei were stained with 5 µg/ml of To-Pro-3 in PBS for 15 min at room temperature. The 3-D structures were mounted under glass coverslips with 0.1% PDD and 90% glycerol in PBS. For immunostaining of laminin V, the fixed 3-D structures were permeabilized with 0.5% Triton X-100 in PBS for 30 min at 4°C and quenched with 100 mM glycine in PBS. Then, the 3-D structures were blocked with buffer A (130 mM NaCl, 7 mM Na2HPO4, 3.5 mM NaH2PO4, 0.1% BSA, 0.2% Triton X-100, 0.05% Tween 20, and 10% normal goat serum) for 2 h and further blocked with buffer B (buffer A plus 20 mg/ml goat anti-mouse F(ab`)2 fragments) for 1 h. The 3-D structures were incubated with anti-laminin V antibody overnight at 4°C. After washed, the 3-D structures were stained with FITC-conjugated secondary antibody for 1 h. The nuclei staining and mounting of slides were performed as above. The images of acinar structures were captured by the Z-stacking function for serial confocal sectioning at 2 µm intervals (LSM-510 Carl Zeiss Laser Scanning Micoroscope) and then analyzed by Carl Zeiss software.

### RNA Electrophoretic Mobility Shift Assay (REMSA)

Various regions in p63 3′ UTR were amplified by PCR with primers containing T7 promoter sequence (5′-GGA TCC TAA TAC GAC TCA CTA TAG GGA G-3′). REMSA was performed as previously described [34]. Briefly, RNA probes were made from *in vitro* transcription by T7 RNA polymerase in the presence of α-^32^P-UTP. REMSA was performed with 200 nM of recombinant protein, 1 mg/ml of yeast tRNA and 50000 CPM ^32^P-labeled RNA probe in a reaction buffer (10 mM Tris-Cl, pH 7.5, 25 mM KCl, 5 mM MgCl_2_, 1 mM DTT) for 20 min at 37°C. RNA/protein complexes were digested with 100 U RNase T1 for 10 min at 37°C and then separated in 6% of native PAGE. RNA-protein complexes were visualized by autoradiography. For supershift assay, 3 µg of anti-HA antibody was pre-incubated with HA-tagged proteins for 30 min on ice prior to incubation with a RNA probe.

### SA-β-galactosidase Staining Assay

This assay was performed as described previously [53]. Cells were washed with phosphate-buffered saline and fixed with 2% formaldehyde and 0.2% glutaraldehyde for 10 min at room temperature. Cells were then washed twice with phosphate-buffered saline and stained with fresh SA-β-galactosidase staining solution at 37°C. The SA-β-galactosidase staining solution contains 1 mg/ml 5-bromo-4-chloro-3-indolyl-β-D-galactopyranoside, 40 mM citric acid/sodium phosphate (pH 6.0), 5 mM potassium ferrocyanide, 5 mM potassium ferricyanide, 150 mM NaCl, and 2 mM MgCl_2_.

### Measurement of Newly Synthesized ΔNp63

To measure newly synthesized ΔNp63 protein, immunoprecipitation was performed with extract from MCF10A-LacZ-KD and MCF10A-HuR-KD cells, which were culture for 24 h, and then labeled with [^35^S]-methionine for 30 min.

### Statistics

All experiments were performed at least in triplicates. Numerical data were expressed as mean ± SD. Two group comparisons were analyzed by two-sided Student's *t* test. *P* values were calculated and *P*<0.05 was considered significant.
